# Physicians' Knowledge, Attitudes, and Perception Toward Pediatric Palliative Care in Saudi Arabia: A National Exploratory Survey

**DOI:** 10.1089/pmr.2023.0010

**Published:** 2023-07-21

**Authors:** Saadiya Khan, Kim Sadler, Khawar Sidiqui, Hamad AlYami, Malak AlGarni, Amani Al-Kofide, Antonello Podda

**Affiliations:** ^1^ Department of Pediatric Hematology/Oncology and King Faisal Specialist Hospital and Research Center, Riyadh, Saudi Arabia.; ^2^ Department of Oncology and Liver Diseases, King Faisal Specialist Hospital and Research Center, Riyadh, Saudi Arabia.

**Keywords:** pediatric palliative care, physician attitude and knowledge, Saudi Arabia

## Abstract

**Background:**

Pediatric palliative care (PPC) helps maintain the quality of life for both children and their families. It has been identified as an important goal within the global health agenda. In Saudi Arabia, the discipline remains in its infancy, as illustrated by the absence of PPC programs in academic and health care institutions.

**Aim:**

The aim was to conduct a pilot study assessing physicians' knowledge, attitudes, and perceptions toward PPC.

**Method:**

Data were gathered through a self-administered questionnaire sent to physicians working in Saudi Arabia.

**Results:**

One hundred twelve completed the survey (male 54.2%, *n* = 50). A total of 40.8% (*n* = 42) had 20 years or more of experience, 42.9% (*n* = 48) were from the hematology-oncology specialty, and 68.5% (*n* = 74) received no training in PPC. Half suggested that children should be informed of their condition but mostly when reaching 12 or 15 years of age. Various physicians reported that the most appropriate time to discuss a transition to palliative care goals is when diagnosing an incurable condition or when despite all efforts, a condition continues to progress and death is expected.

**Conclusion:**

Multiple gaps were identified. PPC basic concepts should be included in the formal medical curriculum (e.g., pain management, communication, and ethical considerations at the end of life). There is also a significant need to develop further both primary and specialized palliative care.

## Introduction

Pediatric palliative care (PPC) is a service provided to children with serious life-limiting or life-threatening diseases such as genetic, neurologic, heart, lung, cancer, or other such chronic conditions with the purpose of reducing their grief and suffering.^1^ According to the World Health Organization (WHO), PPC can assist in improving the quality of life of children and their families by focusing on patient-centeredness and multidisciplinary care as key concepts.^2^ The idea of PPC is to match treatment strategies with the requirements of these children with major illnesses such as pain relief, nausea, constipation, shortness of breath, anxiety, and difficulty sleeping.

Two decades ago, the King Faisal Specialist Hospital & Research Center (Riyadh) established a palliative care center, which gradually increased awareness across the country. At present there are about 12 centers providing such care largely for adults.^3^ Enthusiasm and endorsement worldwide for children to receive these services and its association with potential improvement in their quality of life have led to the recognition of pediatric services in the Kingdom of Saudi Arabia (KSA).^4–7^ Despite the recognition of specialized pediatric palliative services in the developed world, there are still relatively few children in Saudi Arabia and the rest of the developing world who have ready access to these facilities.^8,9^

We believe there is a common misunderstanding about the crossroads between palliative and end-of-life (EoL) care in our current practice. Our local hospitals do not have specialized PPC services. To the best of our knowledge, there are no pediatric inpatient units and/or outpatient clinics offering these dedicated services. At our center, we have now started educational and awareness activities about the necessity of these resources and the benefits that come with it. The major obstacle we face with health care professionals concerns their predominant assumption that palliative care is for dying patients only.

PPC is not only about EoL care, but assists in providing symptom management and guiding parents in decision making for patients with serious illnesses.^10^ We hope for increased education and awareness among health care specialists, patients, and families regarding the scope of pediatric palliative practice with an expansion of PPC programs in academic and health care institutions.^11,12^ This discipline remains in its infancy in the Middle Eastern region.^13,14^ The goal of this exploratory study was to assess physicians' knowledge, attitudes, and perceptions toward PPC in KSA. Herein we examined the confidence in providing palliative care, goals-of-care discussions, communication needs, and perceived barriers to palliative care among a few other measures.

## Methods

### Survey design

This survey questionnaire was developed based on our review of palliative care perception literature and discussion among research team members. A pilot survey was conducted by three pediatric oncology physicians practicing for more than a decade with ample palliative care experience nationally. The survey contained 22 multiple choice and Likert-scale questions on the most salient topics related to PPC such as the attitudes toward EoL discussions, degree of confidence in providing various components of PPC, basic knowledge about opioid use, and barriers limiting access to PPC, including in the home care setting. A brief sociodemographic section was added to the survey with a focus on physicians' training in PPC and experience in caring for children at the EoL. The survey link (Supplementary Data) was sent through e-mails within our institution and to outside hospitals within the KSA at least three times within a period of six months.

### Selection and description of participants

This study was submitted to the Institutional Review Board and was approved by the Research Advisory Committee through established procedures with Approval Number 2211042. All participants consented to take the survey. This survey did not involve patients. Our study was granted approval by the hospital's review board. Thereafter all physicians and trainees taking care of children and adolescents (from birth to 18 years) with an active medical license in the KSA were invited to participate through various channels including but not limited to organizational e-mail invitations, social media platforms, and general e-mail distribution through the Saudi Commission for Health Specialties (SCHS). The survey was active for 12 months from the time of dissemination. All respondents consented to participation.

### Statistical considerations

The data were analyzed using descriptive statistics for participants' demographics and frequency of items. Chi-squared test or Fisher's exact test was used to test for the significance of association between categorical variables, while the Mann–Whitney *U* test and Kruskal–Wallis test were used to test for the significance of difference for composite scores against categorical variables. Multiple correspondence analysis was performed using R package FactoMineR in R environment for statistical computing. All analyses were two-tailed, and differences were statistically significant when *p* was <0.05.

## Results

### Sample characteristics

The survey was sent to more than a thousand physicians within the region. A total of 112 surveys were returned from pediatricians as well as from other subspecialists with no partial surveys submitted. Half of the respondents were male (54.2%, *n* = 50). Most were consultant physicians (54.5%, *n* = 61) and had over 20 years of experience (40.8%, *n* = 42) in the field. Almost half of them were from the pediatric hematology-oncology specialty (42.9%, *n* = 48). Majority of the respondents (68.5%, *n* = 74) had received no formal training in PPC during their initial medical education. Additional sociodemographic data can be found in Table 1.

**Table 1.** Demographics (*N* = 112)

Variables of interest	*n* (%)
Gender	
Female	52 (46.4)
Male	60 (53.6)
Age group (years)	
20–29	9 (8.0)
30–39	33 (29.5)
40–49	37 (33.0)
50 and older	33 (29.5)
Current position	
Medical resident	11 (9.8)
Fellow	16 (14.3)
Assistant consultant	24 (21.4)
Consultant	61 (54.5)
Work sector	
Governmental	100 (89.3)
Private	12 (10.7)
Specialty	
Hematology-oncology	48 (42.9)
Pediatrics	27 (24.1)
Critical care	5 (4.5)
Other specialties	32 (28.5)
Experience^a^	
<5 Years	18 (16.1)
5–10 Years	20 (17.9)
10–20 Years	31 (27.7)
>20 Years	43 (38.4)

^a^ Number of years of experience after finishing medical college.

### Physicians' confidence in providing various types of care

**FIG. 1.**
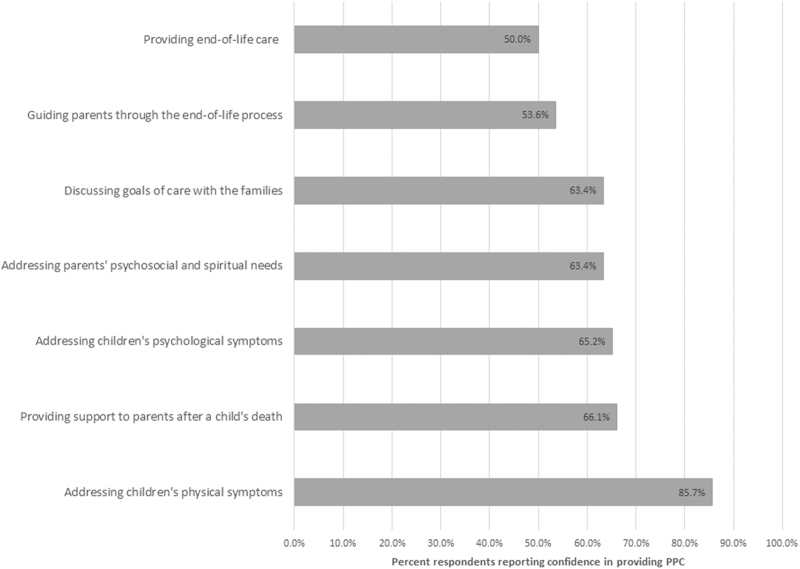
Physician confidence in providing PPC. PPC, pediatric palliative care.

In this survey, physicians felt most confident (confident or very confident) in managing physical symptoms (85.7%, *n* = 96), supporting emotional families immediately after the child's death (66.1%, *n* = 74), and providing emotional support to the child during the illness course (65.2%, *n* = 73). Physicians felt relatively less confident in dealing with situations related to EoL care (50%, *n* = 56) and handling families caring for children with imminent death (53.6%, *n* = 60; Fig. 1). More than a third of the respondents (35%, *n* = 39) reported rarely having contacted the parents after the death of a child, even if they had been actively involved in the care for an extended period.

**FIG. 2.**
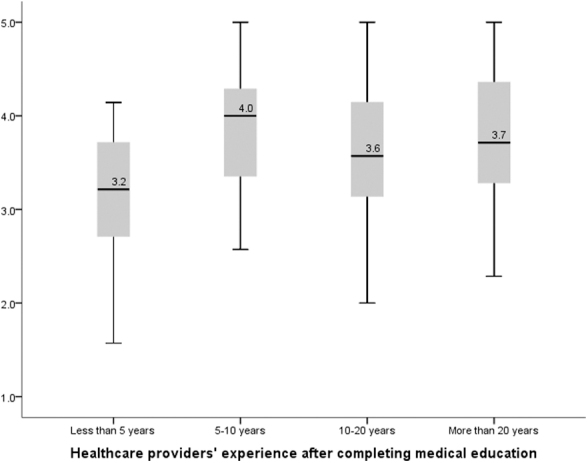
Composite confidence score by physicians' experience.

All the survey questions related to the confidence level in providing various components of PPC such as discussion of palliative goals of care, children's physical and psychological symptoms, providing family education about the process of dying, EoL care, and grief support were averaged to obtain a confidence composite score for each participant. The median overall confidence score was 3.71 (range, 1.57–5.00). It was significantly higher for the physicians who had received PPC training during their formal medical education (4.000 vs. 3.571, *p* = 0.008). The same was also true for the number of years of experience following completion of their medical education (*p* = 0.028; Fig. 2).

### Physicians' knowledge about opioid use

The survey revealed significant gaps in basic knowledge about opioid use. Addiction was reported as being a significant concern while using morphine in children (35.7%, *n* = 40). Injectable morphine was reported to be superior in efficacy to the oral route (37.5%, *n* = 42). A number of physicians reported that opioids should only be prescribed on an as-needed basis (41.1%, *n* = 46) and about 25.9% (*n* = 29) recommended its initiation when pain becomes unbearable.

### Goals-of-care discussion

Majority of physicians responded that the most appropriate time for discussing transition from therapeutic to palliative care was when establishing diagnoses of conditions unlikely to be cured (44.6%, *n* = 50) or when despite all curative efforts, the condition continued to progress and death was expected (46.4%, *n* = 52). When family members insisted to pursue curative treatments despite apparent medical futility, most physicians reacted by repeating the facts about the child's medical condition and emphasized the absence of available curative treatments. Only 29.5% (*n* = 33) responded that they would attempt to explore the meaning of the parent's request. Approximately a third of physicians reported experiencing difficulty conducting EoL care discussions more than once a year.

**FIG. 3.**
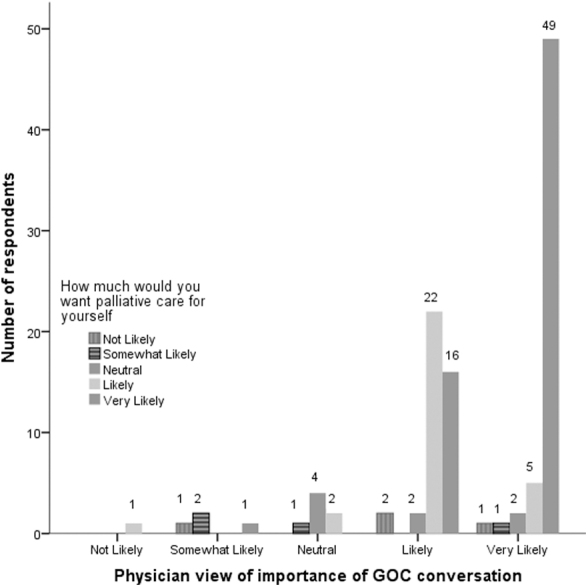
Physician view of importance of GOC conversation.

Multiple correspondence analysis alluded to the underlying structure of questions related to palliative care goals and curative goals. Physicians' perception of goals of care and the choice of palliative care for themselves were also found to be significantly associated with their willingness to discuss the same for their patients (*p* < 0.001; Fig. 3).

### Communication with children

About half of the physicians (46.5%, *n* = 52) responding indicated that children should be made aware of their life-limiting diagnosis. About half of the responders (55.8%, *n* = 29) were in favor of involving children older than 12 years in decision-making discussions. Similar answers were provided regarding the disclosure of prognosis to children. A few reasons for physicians being hesitant in disclosing information to children included beliefs that it could cause great emotional shock to the child (39.2%, *n* = 20), parents should be the only ones informed (29.4%, *n* = 15), and the fear that the child would lose hope (19.6%, *n* = 10).

No significant association was observed between the physicians' experience with children at the EoL and their level of difficulty in discussing do-not-attempt-resuscitation (*p* = 0.284).

**FIG. 4.**
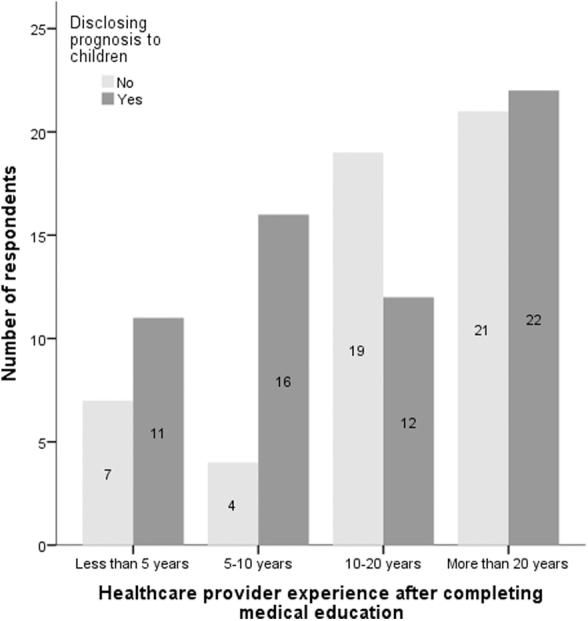
Disclosing prognosis to children.

Moreover, physicians' confidence in discussing prognosis with children was significantly associated with the number of years of experience (*p* = 0.031), revealing that more experience was associated with more open discussions. We saw a higher trend in young physicians, most likely due to more exposure, training, and open dialogue in the recent past (Fig. 4).

### EoL care at home

Although the majority of physicians recognized that children would prefer to spend their EoL at home (67%, *n* = 75), multiple barriers were identified. Physicians identified the following barriers to providing EoL care home: families' anxiety to deal with death at home (84.8%, *n* = 95), emotional impact on their other children (71.4%, *n* = 80), lack of resources at home (59.8%, *n* = 67), and suboptimal communication between families and treating team about goals of care and preferences at the EoL (51.8%, *n* = 58).

### Barriers to PPC delivery in Saudi Arabia

**FIG. 5.**
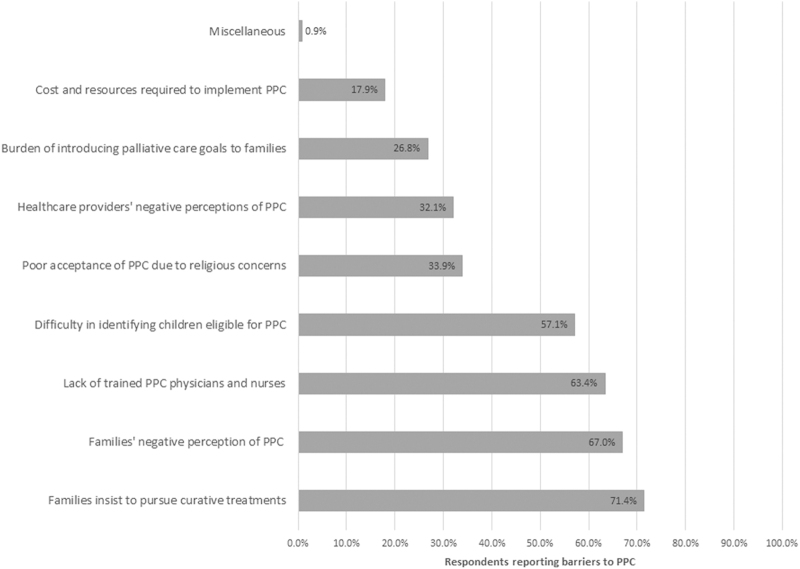
Respondents reporting barriers to PPC.

Families' preference to pursue curative treatments was identified as the most important barrier (71.4%, *n* = 80), followed by the negative implication that PPC has for parents (67%, *n* = 75), as well as the lack of trained physicians and nurses (63.4%, *n* = 71) (Fig. 5). Physicians' perception regarding family's concerns about disturbance caused at home, being scared of handling death, lack of resources, and suboptimal communication between the family and the treating team were identified as significant barriers (*p* < 0.001).

### Physicians' stress and coping mechanisms

A third of the physicians (33.8%, *n* = 49) reported experiencing stress often when dealing with the death of a child. The most commonly used coping mechanisms included praying (60.7%, *n* = 68), talking with work colleagues (60.7%, *n* = 68), and chatting with friends and family members (53.6%, *n* = 60). Significant association was reported by the respondents between praying while being in stress due to the loss of their patients (*p* < 0.001).

## Discussion

In this cross-sectional survey, we examined the confidence in discussing goals of care, ease of communication with children, as well as EoL care followed by the perceived barriers to the kingdom-wide implementation of PPC services. Our survey results showed that physicians felt more confident in dealing with physical symptoms. Studies have demonstrated the benefit of PPC partnership along with the primary team, toward improvement in several psychosocial outcomes during a serious clinical course.^15,16^ In children with cancer, PPC integration has been linked with better management, and hence, the need for broader application of this specialized service for other prevalent and widespread life-threatening illnesses within the region.^17,18^

Gaps in opioid knowledge is a worrisome finding as these can contribute significantly to inadequate treatment of pain.^19^ Children with life-threatening conditions are particularly prone to experience chronic and acute pain from the disease, accompanying investigations, and/or the various treatment modalities that are administered. A prospective study conducted in children with advanced cancer revealed that nearly 40% had a highly distressing level of pain, with pain reported by nearly 60% of children at the EoL.^20^ Pain is also common in many noncancer conditions, such as neurologic and metabolic conditions with their subsequent clinical deterioration. It is interesting to see that despite the importance of pain and it being considered the fifth vital sign, only a third of our responding physicians (31.3%, *n* = 35) had received any formal education about pain and PPC learning during their initial medical education.^21,22^

In a study examining the experience of bereavement in parents about EoL communication, families expressed the need for ongoing and open communication about their child's prognosis.^23^ Medical team members suffer significant emotional stress while engaging in difficult discussions.^24^ A third of our physicians reported anxiety and worry related to these tough situations. Given such reports, we believe that all trainee physicians involved with palliative care are susceptible to acute anxiety and tension after a patient's death. All engaged staff should have available to them additional coping resources and support.^25^ Future research projects need to study the effects of behavioral and educational intervention for staff along with an assessment of enhanced coping strategies.^26,27^

Despite avoiding palliative care discussions with children, most of them seem to be aware that something is wrong.^28^ Just like adults, children have worries and when left unaddressed, these can become more intense.^29^ Younger children are most likely to be distressed by medical procedures, admissions, and physical symptoms, while older ones tend to be more affected by emotional, psychological, and social suffering.^30^ About half of our respondents indicated that children should be made aware of their life-limiting diagnosis. Those who were not in favor thought that disclosing information would discourage and emotionally be tiring for children. Communication with children is encouraged by all, but it should be adapted according to their level of understanding depending on the developmental age and emotional state. Integration of specialized PPC team can assist with these challenging communication scenarios and assist parents and primary physicians who may be more hesitant to speak with children.

Generally, in our part of the world, health care providers and organizational leaders lack awareness regarding the scope of PPC and its importance in the holistic care of children. This is evident from the number of responders to this survey given that physicians taking care of children and adolescents are way more than those who answered. Even in settings where PPC programs exist, there are barriers to their optimal potential given the lack of support and human resources.^31^ There was a reluctance to refer mainly due to inadequate screening, unavailability of services, as well as fear from primary teams that families would not be able to handle death at home.^32^ Such barriers lead to either absent or late referrals to specialized PPC services. These obstacles need to be overcome. Early referrals are associated with a reduction in stress and suffering while also being cost-effective.^13,33,34^

A cross-sectional study design and a limited respondent size preclude the finding of any causal association. This study can be considered an initial pilot sampling survey. Another limitation of this survey is selection bias, possibly due to those physicians responding who considered PPC important and maybe even had more knowledge as well as experience with this subspecialty.

In conclusion, we need to consolidate our resources, personnel, and implement laws to improve the quality of life in seriously ill children. Future studies should also directly survey children and their families to assess if families truly feel uncomfortable with having children die at home and the impact it has on their social and psychological well-being. Home health care option can be considered if available and acceptable by the families in accordance with their child's preference.

## Supplementary Material

Supplemental data

## Abbreviations Used

EOL: end of life
KSA: Kingdom of Saudi Arabia
PPC: pediatric palliative care
