# Supplementary material for: Physicians' Knowledge, Attitudes, and Perception Toward Pediatric Palliative Care in Saudi Arabia: A National Exploratory Survey
Source: Palliat Med Rep. 2023 Jul 21;4(1):185–92. doi: 10.1089/pmr.2023.0010 (PMC10366288; doi:10.1089/pmr.2023.0010)
Supplement: Supplemental data [file Supp_Data.pdf]

Pediatric & Adolescent Palliative Care Awareness Survey

Goals of Care Definition

We define communication and decision-making about goals of care as a conversation in which, ideally, a patient or family member and the healthcare team establish the goals of treatment together (e.g., cure, prolongation of life, comfort) and agree upon the types of life-sustaining technology that will (or will not) be used to achieve those goals (e.g., CPR, mechanical ventilation, dialysis, intensive care unit admission, feeding tubes, or intravenous hydration).

PALLIATIVE CARE GOALS OF CARE

When is the most appropriate timing to initiate discussions about transitioning to palliative goals of care with a child's family? (Select the most appropriate answer)

- ○ When diagnosing a disease that is unlikely to be curable (Eg. Duchenne muscular dystrophy, progressive metabolic disorder, glioblastoma)
- ○ When despite all curative efforts, the child's condition continues to deteriorate and death is expected
- ○ When death is expected within the next year
- ○ When death is expected within the next 3 to 6 months
- ○ When death is expected within days or weeks
- ○ Palliative care goals should not be openly discussed with family

As a physician, if your patient has a terminal illness, unlikely to be cured and with a poor prognosis, how likely are you pursue the following goals of care?

	Not Likely	Somewhat Likely	Neutral	Likely	Very Likely
Pursue with life-sustaining treatments (eg. Intensive Care Unit, ventilator, CPR, inotropes, dialysis)	○	○	○	○	○
Engage with child and/or family in discussions about transitioning to palliative goals of care (eg. focus on comfort care)	○	○	○	○	○

Hypothetically, if you were diagnosed with a terminal illness, unlikely to be cured and with a poor prognosis, to which extent would pursue these following goals of care for yourself?

	Not Likely	Somewhat Likely	Neutral	Likely	Very Likely
Pursue with life-sustaining treatments (eg. Intensive Care Unit, ventilator, CPR, inotropes, dialysis)	○	○	○	○	○
Transition to palliative goals of care (eg. focus on comfort care)	○	○	○	○	○

SITUATION: A child has a serious illness with no cure possible and you feel that disease-modifying treatments and aggressive measures (eg. CPR) would be futile, but the patient's family is strongly requesting that "everything be done".

What is your usual first reaction?

◯ I spend time re-explaining the facts around the condition and the absence of disease-modifying treatments.
◯ I attempt to understand better the meaning of the request.
◯ I do not accept their request and transition the care to palliative goals of care.
◯ I accept their request and pursue with disease-modifying treatments.

How often do you experience difficulties with changing code status (DNR) and convincing parents that there are no more curative options?

◯ Almost never
◯ More than once a year
◯ More than once every 6 months
◯ More than once every 3 months
◯ More than once a month

DIAGNOSIS DISCLOSURE TO CHILDREN

Do you think that physicians should DISCLOSE potentially life-limiting DIAGNOSES to children and teenagers?

◯ Yes
◯ No

From which age is it appropriate to DISCLOSE a potentially life-limiting DIAGNOSIS to children and teenagers?

◯ 4 years old and older
◯ 7 years of age and older (eg. elementary school students or older)
◯ 12 years old and older (eg. junior high school students)
◯ 15 years old and older (eg. high school & college students)

Do you think physicians should involve children and teenagers in decision-making regarding treatments once they are aware of their DIAGNOSIS?

◯ Yes
◯ No

PROGNOSIS DISCLOSURE TO CHILDREN

Do you think that physicians should DISCLOSE information about DISEASE PROGRESSION and PROGNOSIS to children and teenagers?

- ◯ Yes
- ◯ No

From which age is it appropriate to DISCLOSE information about disease progression and PROGNOSIS to children and teenagers?

- ◯ 4 years old and older
- ◯ 7 years of age and older (eg. elementary school students or older)
- ◯ 12 years old and older (eg. junior high school students)
- ◯ 15 years old and older (eg. high school & college students)

Why would you be reluctant to disclose a life-limiting illnesses to a child or teenager? (Select the most important reason)

- ◯ I am afraid the child would be discouraged and lose hope.
- ◯ I do not think it is necessary to tell a child about such diagnosis.
- ◯ I think the child's condition may worsen due to the psychological shock of the news.
- ◯ I believe that the decision-makers (parents) should be the only ones informed.

Do you think physicians should involve children and teenagers in decision-making regarding treatments once they are aware of their PROGNOSIS?

- ◯ Yes
- ◯ No

END OF LIFE CARE

How frequently are you taking care of children who are considered at end-of-life (last weeks or days of life)?

- ◯ Never
- ◯ Rarely
- ◯ Sometimes
- ◯ Frequently
- ◯ Very Often

Approximately how many of your pediatric patients have died in the last 12 months?

- ◯ None
- ◯ 1-10
- ◯ 11-20
- ◯ 21 or more

PROVIDING PALLIATIVE CARE

Rate your degree of confidence in your abilities to engage in these tasks:

	No Confidence	Some Confidence	Neutral	Confident	Very Confident
Discussing palliative goals of care	○	○	○	○	○
Addressing children's physical symptoms (eg. pain, dyspnea, nausea)	○	○	○	○	○
Addressing children's psychological symptoms (eg. anxiety, sadness, anger)	○	○	○	○	○
Addressing psychosocial and spiritual needs with parents (eg. dealing with their denial, preparing them for the end of life)	○	○	○	○	○
Providing family education about the process of dying when the child approaches the end of life (eg. signs to expect, care adjustment)	○	○	○	○	○
Providing end of life care to children (eg. last days or hours)	○	○	○	○	○
Providing emotional support to the parents once the child has died (eg. during death declaration, after death)	○	○	○	○	○

To what extent do you agree with the following statements in reference to the use of opioids like morphine?

	Strongly Agree	Agree	Neither Agree Nor Disagree	Disagree	Strongly Disagree
Morphine is addictive.	○	○	○	○	○
Morphine in injectable form is better than in the oral form.	○	○	○	○	○
Once morphine is started, death is near.	○	○	○	○	○
Morphine lowers breathing function.	○	○	○	○	○
Morphine should only be used on an as needed basis.	○	○	○	○	○
Morphine should be prescribed only when the pain is really unbearable.	○	○	○	○	○

In your experience where do most of your pediatrics children would prefer to spend their last days of life?

○ At home
○ In the hospital

In your experience what barriers do you face in discharging patients with no curative options while waiting for death?

	Strongly Agree	Agree	Neither Agree Nor Disagree	Disagree	Strongly Disagree
Family is not keen on bringing patients home because they are scared of handling death at home.	○	○	○	○	○
Family are often worried that there are other children at home that will get disturbed.	○	○	○	○	○
Patients are trying to avoid being a burden to their family.	○	○	○	○	○
There is suboptimal communication between patient family and treating team regarding goals of care.	○	○	○	○	○
There is a lack of resources to discharge patients at home such as oxygen, IV fluids, medication administration at home	○	○	○	○	○

SITUATION: If a patient of yours that you have been following for a prolonged period died in your absence, do you contact the parents at some point to offer your sympathy and see how they are coping?

- ◯ This is not part of my actual practice
- ◯ Rarely
- ◯ Sometimes
- ◯ Most of the time
- ◯ Always

Are you experiencing psychological stress (eg. tired, angry, and emotionally drained, compassion fatigue) while dealing with the death of your pediatric patients?

- ◯ Never
- ◯ Rarely
- ◯ Sometimes
- ◯ Often
- ◯ Very Often

What are your coping strategies to deal with this stress? (Select all answers that apply)

- ☐ Music
- ☐ Exercise
- ☐ Praying
- ☐ Reading
- ☐ Yoga
- ☐ Talking with colleagues
- ☐ Talking with friends or family members
- ☐ Others

Other

SPECIALIZED PALLIATIVE CARE SERVICES

What are the most significant barriers towards pediatric palliative care delivery in Saudi Arabia? (Select all that apply)

- ☐ Attitude of patients and families who often want to pursue curative treatments as far as possible.
- ☐ Negative perceptions of patients and families about the palliative care approach (e.g. may lead to an early death, means giving up on patients).
- ☐ Negative perceptions of the medical personnel (e.g. may lead to an early death, means giving up on patients)
- ☐ Difficulty in identifying patients who can benefit from palliative care.
- ☐ Uncertainty about when to refer to palliative care (uncertainty about disease prognosis, treatment response timing)
- ☐ Burden of introducing palliative care goals to patients & their families.
- ☐ Concerns on additional cost, time and effort required to develop palliative care programs.
- ☐ Lack of trained palliative care doctors and nurses
- ☐ Religious acceptance of a palliative care approach
- ☐ Others

Others:

SOCIO-DEMOGRAPHIC INFORMATION

What is your gender?

◯ Male
◯ Female

Which age category are you in?

◯ 20-29
◯ 30-39
◯ 40-49
◯ 50 and over

Did you complete all or a part of your medical education outside Saudi Arabia?

◯ Yes
◯ No

Have you ever received training about pediatric palliative care and/or medical decision-making at the end of life? (Select all answers that apply)

☐ During my formal medical education
☐ In Hospital training
☐ Conference(s) where there were presentations about palliative care
☐ Self-learning (e.g. online module, reading articles)
☐ I have no training in palliative care

What is your current position?

◯ Medical Resident
◯ Fellow
◯ Assistant Consultant
◯ Consultant

What is your current practicing specialty?

How many years of experience do you have since completing your medical education (after medical college)?

◯ Less than 5 years
◯ 5 to 10 years
◯ 10 to 20 years
◯ More than 20 years
